# Diammine{*N*-[2-(hy­droxy­imino)­propion­yl]-*N*′-[2-(oxidoimino)­propion­yl]propane-1,3-diaminido-κ^4^ *N*,*N*′,*N*′′,*N*′′′}iron(III)

**DOI:** 10.1107/S160053681204826X

**Published:** 2012-11-30

**Authors:** Stefania Tomyn, Matti Haukka, Ruslan Nedelkov

**Affiliations:** a Department of Chemistry, National Taras Shevchenko University of Kyiv, Volodymyrska Street 64, 01601 Kyiv, Ukraine; b Department of Chemistry, University of Jyvãskylã, PO Box 35, FI-40014, Finland

## Abstract

In the title compound, [Fe(C_9_H_13_N_4_O_4_)(NH_3_)_2_], the Fe^III^ atom, lying on a mirror plane, is coordinated by four N atoms of a triply deprotonated tetra­dentate *N*-[2-(hy­droxy­imino)­propion­yl]-*N*′-[2-(oxidoimino)­propion­yl]propane-1,3-diaminide ligand in the equatorial plane and two N atoms of two ammonia mol­ecules at the axial positions in a distorted octa­hedral geometry. A short intra­molecular O—H⋯O hydrogen bond between the *cis*-disposed oxime O atoms stabilizes the pseudo-macrocyclic configuration of the ligand. In the crystal, mol­ecules are linked by N—H⋯O hydrogen bonds into a three-dimensional network. The ligand has a mirror-plane symmetry. One of the methyl­ene groups of the propane bridge is disordered over two sets of sites with equal occupancy factors.

## Related literature

For oximes as potential bridging ligands, see: Moroz *et al.* (2008 ▶, 2010 ▶); Skopenko *et al.* (1990 ▶). For oximes stabilizing high oxidation states of metal ions, see: Fritsky *et al.* (1998 ▶, 2006 ▶); Kanderal *et al.* (2005 ▶). For the coordination chemistry of tetradentate open-chain ligands derived from oximes and amides, see: Duda *et al.* (1997 ▶); Fritsky *et al.* (2004 ▶); Kufelnicki *et al.* (2010 ▶). For related structures, see: Dvorkin *et al.* (1990*a* ▶,*b* ▶); Lampeka *et al.* (1989 ▶); Mokhir *et al.* (2002 ▶); Onindo *et al.* (1995 ▶); Sliva *et al.* (1997*a* ▶,*b* ▶).
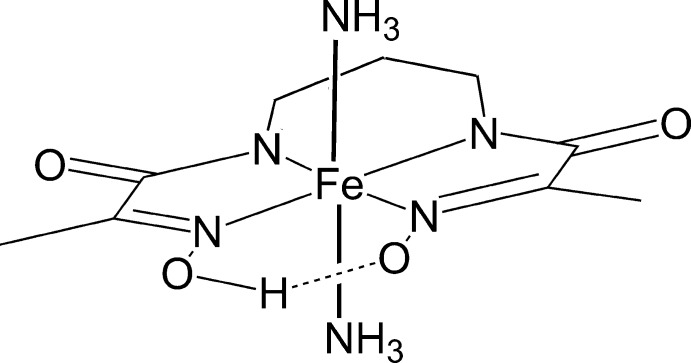


## Experimental

### 

#### Crystal data

[Fe(C_9_H_13_N_4_O_4_)(NH_3_)_2_]
*M* *_r_* = 331.15
Monoclinic, 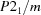

*a* = 8.9111 (3) Å
*b* = 7.2255 (3) Å
*c* = 10.6194 (4) Å
β = 108.994 (2)°
*V* = 646.52 (4) Å^3^
*Z* = 2
Mo *K*α radiation
μ = 1.19 mm^−1^
*T* = 100 K
0.20 × 0.09 × 0.04 mm

#### Data collection

Nonius KappaCCD diffractometer
Absorption correction: multi-scan (*DENZO*/*SCALEPACK*; Otwinowski & Minor, 1997 ▶) *T*_min_ = 0.795, *T*_max_ = 0.954
12359 measured reflections
1599 independent reflections
1361 reflections with *I* > 2σ(*I*)
*R*_int_ = 0.047

#### Refinement

*R*[*F* ^2^ > 2σ(*F* ^2^)] = 0.030
*wR*(*F* ^2^) = 0.074
*S* = 1.04
1599 reflections
124 parameters
H-atom parameters constrained
Δρ_max_ = 0.58 e Å^−3^
Δρ_min_ = −0.46 e Å^−3^

### 

Data collection: *COLLECT* (Nonius, 2000 ▶); cell refinement: *DENZO*/*SCALEPACK* (Otwinowski & Minor, 1997 ▶); data reduction: *DENZO*/*SCALEPACK*; program(s) used to solve structure: *SHELXS97* (Sheldrick, 2008 ▶); program(s) used to refine structure: *SHELXL97* (Sheldrick, 2008 ▶); molecular graphics: *XP* in *SHELXTL* (Sheldrick, 2008 ▶) and *Mercury* (Macrae *et al.*, 2006 ▶); software used to prepare material for publication: *SHELXL97*.

## Supplementary Material

Crystal structure: contains datablock(s) global, I. DOI: 10.1107/S160053681204826X/hy2607sup1.cif

Structure factors: contains datablock(s) I. DOI: 10.1107/S160053681204826X/hy2607Isup2.hkl

Additional supplementary materials:  crystallographic information; 3D view; checkCIF report

## Figures and Tables

**Table 1:** Hydrogen-bond geometry (Å, °)

*D*—H⋯*A*	*D*—H	H⋯*A*	*D*⋯*A*	*D*—H⋯*A*
O1—H1*O*⋯O4	0.98	1.54	2.508 (3)	168
N5—H5*D*⋯O4^i^	0.91	2.15	3.015 (2)	158
N5—H5*E*⋯O2^ii^	0.91	2.11	2.979 (2)	160
N5—H5*F*⋯O3^iii^	0.91	2.10	2.969 (2)	160

Symmetry codes: (i) 
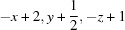
; (ii) 
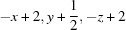
; (iii) 
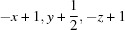
.

**Fig. 1.**
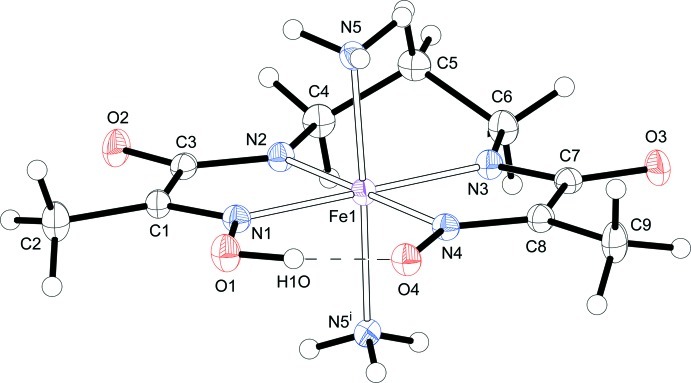
The molecular structure of the title compound. Displacement ellipsoids are drawn at the 50% probability level. C5 atom and all C-bound H atoms are disordered over two sets of sites across the mirror plane with equal occupancies. [Symmetry code: (i) x, 3/2-y, z.]

**Fig. 2.**
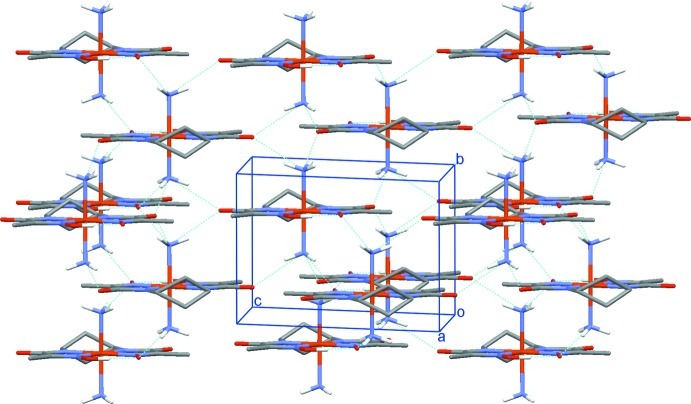
A packing diagram of the title compound. Hydrogen bonds are indicated by dashed lines. H atoms not involved in hydrogen bonds have been omitted for clarity.

## supplementary crystallographic information

### Comment

Polydentate oxime ligands containing both oxime and other donor functions (*e.g.* carboxylic, amide, hydroxamic) draw considerable attention during past two decades due to their potential for the bridging mode of coordination and mediation of strong magnetic exchange interactions between metal ions (Moroz *et al.*, 2008, 2010; Skopenko *et al.*, 1990) and for the preparation of metal complexes with efficient stabilization of unusually high oxidation states of 3d-metal ions (Fritsky *et al.*, 1998, 2006; Kanderal *et al.*, 2005). The open-chain tetradentate oxime-and-amide ligands were the subjects of several studies carried out in our group, and a series of mono- and polynuclear complexes of copper(III), nickel(II) and cobalt(III) have been reported within past 15 years (Duda *et al.*, 1997; Fritsky *et al.*, 2004, 2006; Kanderal *et al.*, 2005; Kufelnicki *et al.*, 2010). As a part of our research study of open-chain tetradentate oxime-and-amide ligands, we present the structure of the title compound containing iron(III) as a central atom.

In the title compound, Fe^III^ ion, lying on a mirror plane, is coordinated by four N atoms of a triply deprotonated tetradentate ligand, *N*,*N*'-bis(2-hydroxyiminopropionyl)propane-1,3-diamine, in the equatorial plane and by two N atoms of two ammonia molecules at the axial postions in a distorted octahedral geometry (Fig. 1). The tetradentate ligand coordinates the Fe^III^ atom in a planar fashion, forming three condensed 5-, 6- and 5-membered chelate rings. All angles around the Fe^III^ atom deviate insignificantly from 90°. There are no alternating deviations for N(oxime) and N(amide) atoms from the N1, N2, N3 and N4 plane. The values of Fe—N(amide) and Fe—N(oxime) bond lengths in the equatorial plane are in the range of 1.909 (2)–1.917 (2) Å. The distance between the Fe^III^ atom and the two axial N atom is 1.993 (3) Å. Bond lengths of N—C and C═O of the amide groups are 1.323 (3)–1.327 (3) and 1.252 (3)–1.255 (3) Å, respectively, and are typical for the deprotonated amide groups (Dvorkin *et al.*, 1990*a*, *b*; Lampeka *et al.*, 1989; Onindo *et al.*, 1995). The bond lengths N—O and C═N of the oxime groups are 1.349 (3)–1.364 (3) and 1.291 (3)–1.292 (3) Å, respectively, that is typical for the amide derivatives of 2-hydroxypropanoic acid (Duda *et al.*, 1997; Mokhir *et al.*, 2002; Onindo *et al.*, 1995; Sliva *et al.*, 1997*a*, *b*). In the crystal, the complex molecules are linked by intermolecular N—H···O hydrogen bonds formed between the coordinated ammonia molecules and O atoms of the ligand into a three-dimensional network (Fig. 2). An intramolecular O—H···O hydrogen bond is also present. One of the methylene groups of the propane bridge is disordered in a 0.5:0.5 ratio.

### Experimental

Fe(ClO_4_).6H_2_O (0.363 g, 1 mmol) was dissolved in 5 ml of DMSO and added to a solution of *N*,*N*'-bis(2-hydroxyiminopropionyl)propane-1,3-diamine (0.246 g, 1 mmol) in 10 ml of DMSO. The resulting deep orange mixture was stirred for 15 min at room temperature, filtered off and set aside for crystallization at ambient conditions in an ammonia atmosphere. Red insoluble crystals of the title compound suitable for X-ray analysis were obtained in 24 h (yield: 0.227 g, 68%). Analysis, calculated for C_9_H_19_FeN_6_O_4_: C 39.27, H 6.96, N 30.53%; found: C 39.59, H 7.02, N 30.84%.

### Refinement

C5 atom and all C-bound H atoms were disordered over two sets of sites across the mirror plane, with equal occupancies. These H atoms were positioned geometrically and refined as riding atoms, with C—H = 0.99 (CH_2_) and 0.98 (CH_3_) Å and *U*_iso_(H) = 1.2(1.5 for methyl)*U*_eq_(C). H atoms of OH and NH_3_ groups were located from a difference Fourier map and constrained to ride on their parent atoms, with *U*_iso_(H) = 1.5*U*_eq_(parent atom).

### Crystal data

**Table:**

[Fe(C_9_H_13_N_4_O_4_)(NH_3_)_2_]	*F*(000) = 346.0
*M**_r_* = 331.15	*D*_x_ = 1.701 Mg m^−^^3^
Monoclinic, *P*2_1_/*m*	Mo *K*α radiation, λ = 0.71073 Å
Hall symbol: -P 2yb	Cell parameters from 2700 reflections
*a* = 8.9111 (3) Å	θ = 1.0–27.5°
*b* = 7.2255 (3) Å	µ = 1.19 mm^−^^1^
*c* = 10.6194 (4) Å	*T* = 100 K
β = 108.994 (2)°	Block, red
*V* = 646.52 (4) Å^3^	0.20 × 0.09 × 0.04 mm
*Z* = 2	

### Data collection

**Table:**

Nonius KappaCCD diffractometer	1599 independent reflections
Radiation source: fine-focus sealed tube	1361 reflections with *I* > 2σ(*I*)
Horizontally mounted graphite crystal monochromator	*R*_int_ = 0.047
Detector resolution: 9 pixels mm^-1^	θ_max_ = 27.5°, θ_min_ = 2.4°
φ and ω scans with κ offset	*h* = −11→11
Absorption correction: multi-scan (*DENZO*/*SCALEPACK*; Otwinowski & Minor, 1997)	*k* = −9→9
*T*_min_ = 0.795, *T*_max_ = 0.954	*l* = −13→13
12359 measured reflections	

### Refinement

**Table:**

Refinement on *F*^2^	Primary atom site location: structure-invariant direct methods
Least-squares matrix: full	Secondary atom site location: difference Fourier map
*R*[*F*^2^ > 2σ(*F*^2^)] = 0.030	Hydrogen site location: inferred from neighbouring sites
*wR*(*F*^2^) = 0.074	H-atom parameters constrained
*S* = 1.04	*w* = 1/[σ^2^(*F*_o_^2^) + (0.028*P*)^2^ + 0.7141*P*] where *P* = (*F*_o_^2^ + 2*F*_c_^2^)/3
1599 reflections	(Δ/σ)_max_ < 0.001
124 parameters	Δρ_max_ = 0.58 e Å^−^^3^
0 restraints	Δρ_min_ = −0.46 e Å^−^^3^

### Special details

**Table:**

Geometry. All e.s.d.'s (except the e.s.d. in the dihedral angle between two l.s. planes) are estimated using the full covariance matrix. The cell e.s.d.'s are taken into account individually in the estimation of e.s.d.'s in distances, angles and torsion angles; correlations between e.s.d.'s in cell parameters are only used when they are defined by crystal symmetry. An approximate (isotropic) treatment of cell e.s.d.'s is used for estimating e.s.d.'s involving l.s. planes.
Refinement. Refinement of *F*^2^ against ALL reflections. The weighted *R*-factor *wR* and goodness of fit *S* are based on *F*^2^, conventional *R*-factors *R* are based on *F*, with *F* set to zero for negative *F*^2^. The threshold expression of *F*^2^ > σ(*F*^2^) is used only for calculating *R*-factors(gt) *etc*. and is not relevant to the choice of reflections for refinement. *R*-factors based on *F*^2^ are statistically about twice as large as those based on *F*, and *R*- factors based on ALL data will be even larger.

### Fractional atomic coordinates and isotropic or equivalent isotropic displacement parameters (Å^2^)

**Table:**

	*x*	*y*	*z*	*U*_iso_*/*U*_eq_	Occ. (<1)
Fe1	0.84000 (4)	0.7500	0.68148 (4)	0.01316 (12)	
O1	1.1762 (2)	0.7500	0.72129 (19)	0.0199 (4)	
H1O	1.1180	0.7500	0.6258	0.030*	
O2	0.9831 (2)	0.7500	1.07989 (18)	0.0220 (4)	
O3	0.4412 (2)	0.7500	0.36943 (19)	0.0196 (4)	
O4	0.9952 (2)	0.7500	0.48443 (18)	0.0162 (4)	
N5	0.85135 (18)	1.0255 (2)	0.67972 (15)	0.0151 (3)	
H5D	0.9063	1.0618	0.6252	0.023*	
H5E	0.9016	1.0674	0.7637	0.023*	
H5F	0.7514	1.0730	0.6494	0.023*	
N1	1.0616 (3)	0.7500	0.7808 (2)	0.0148 (4)	
N2	0.8273 (3)	0.7500	0.8580 (2)	0.0149 (4)	
N3	0.6183 (3)	0.7500	0.5839 (2)	0.0146 (4)	
N4	0.8536 (2)	0.7500	0.5050 (2)	0.0135 (4)	
C1	1.1043 (3)	0.7500	0.9092 (3)	0.0166 (5)	
C2	1.2708 (3)	0.7500	0.9983 (3)	0.0243 (6)	
H2A	1.2741	0.7215	1.0894	0.036*	0.50
H2B	1.3177	0.8722	0.9964	0.036*	0.50
H2C	1.3310	0.6563	0.9681	0.036*	0.50
C3	0.9631 (3)	0.7500	0.9576 (3)	0.0162 (5)	
C4	0.6775 (3)	0.7500	0.8870 (3)	0.0191 (6)	
H4A	0.6940	0.8119	0.9736	0.023*	0.50
H4B	0.6456	0.6205	0.8952	0.023*	0.50
C5	0.5460 (4)	0.8461 (6)	0.7819 (4)	0.0181 (8)	0.50
H5A	0.5811	0.9733	0.7708	0.022*	0.50
H5B	0.4534	0.8564	0.8136	0.022*	0.50
C6	0.4926 (3)	0.7500	0.6449 (3)	0.0187 (6)	
H6A	0.4618	0.6207	0.6555	0.022*	0.50
H6B	0.3981	0.8145	0.5852	0.022*	0.50
C7	0.5789 (3)	0.7500	0.4522 (3)	0.0152 (5)	
C8	0.7221 (3)	0.7500	0.4066 (3)	0.0152 (5)	
C9	0.7113 (3)	0.7500	0.2641 (3)	0.0229 (6)	
H9A	0.7349	0.8742	0.2386	0.034*	0.50
H9B	0.6038	0.7142	0.2092	0.034*	0.50
H9C	0.7880	0.6616	0.2505	0.034*	0.50

### Atomic displacement parameters (Å^2^)

**Table:**

	*U*^11^	*U*^22^	*U*^33^	*U*^12^	*U*^13^	*U*^23^
Fe1	0.01225 (19)	0.0146 (2)	0.01244 (19)	0.000	0.00372 (14)	0.000
O1	0.0132 (9)	0.0290 (11)	0.0181 (10)	0.000	0.0062 (8)	0.000
O2	0.0250 (10)	0.0279 (11)	0.0107 (9)	0.000	0.0027 (8)	0.000
O3	0.0121 (9)	0.0254 (11)	0.0183 (10)	0.000	0.0006 (7)	0.000
O4	0.0119 (8)	0.0212 (10)	0.0174 (9)	0.000	0.0073 (7)	0.000
N5	0.0155 (7)	0.0153 (8)	0.0139 (7)	0.0005 (6)	0.0041 (6)	0.0013 (6)
N1	0.0135 (10)	0.0150 (11)	0.0162 (11)	0.000	0.0052 (8)	0.000
N2	0.0150 (10)	0.0172 (11)	0.0131 (10)	0.000	0.0052 (8)	0.000
N3	0.0128 (10)	0.0175 (11)	0.0142 (11)	0.000	0.0053 (8)	0.000
N4	0.0120 (10)	0.0139 (11)	0.0153 (11)	0.000	0.0053 (8)	0.000
C1	0.0172 (13)	0.0147 (13)	0.0147 (12)	0.000	0.0007 (10)	0.000
C2	0.0201 (14)	0.0317 (17)	0.0174 (14)	0.000	0.0011 (11)	0.000
C3	0.0201 (13)	0.0128 (12)	0.0134 (12)	0.000	0.0025 (10)	0.000
C4	0.0181 (13)	0.0229 (14)	0.0181 (13)	0.000	0.0086 (11)	0.000
C5	0.0158 (17)	0.020 (2)	0.0206 (19)	0.0000 (15)	0.0091 (15)	0.0001 (15)
C6	0.0135 (12)	0.0250 (15)	0.0187 (13)	0.000	0.0067 (10)	0.000
C7	0.0147 (12)	0.0127 (12)	0.0174 (12)	0.000	0.0042 (10)	0.000
C8	0.0153 (12)	0.0157 (12)	0.0146 (12)	0.000	0.0048 (10)	0.000
C9	0.0190 (14)	0.0350 (17)	0.0124 (13)	0.000	0.0022 (11)	0.000

### Geometric parameters (Å, º)

**Table:**

Fe1—N3	1.909 (2)	N4—O4^i^	1.349 (3)
Fe1—N1	1.912 (2)	C1—C2	1.478 (4)
Fe1—N2	1.914 (2)	C1—C3	1.506 (4)
Fe1—N4	1.917 (2)	C2—H2A	0.9800
Fe1—N5	1.9932 (16)	C2—H2B	0.9800
O1—N1	1.364 (3)	C2—H2C	0.9800
O1—H1O	0.9769	C4—C5	1.500 (4)
O2—C3	1.252 (3)	C4—H4A	0.9900
O3—C7	1.255 (3)	C4—H4B	0.9900
O4—O4^i^	0.000 (4)	C5—C6	1.541 (4)
O4—N4	1.349 (3)	C5—H5A	0.9900
N5—H5D	0.9100	C5—H5B	0.9900
N5—H5E	0.9100	C6—H6A	0.9900
N5—H5F	0.9100	C6—H6B	0.9900
N1—C1	1.291 (3)	C7—C8	1.505 (4)
N2—C3	1.323 (3)	C8—C9	1.485 (4)
N2—C4	1.464 (3)	C9—H9A	0.9800
N3—C7	1.327 (3)	C9—H9B	0.9800
N3—C6	1.465 (3)	C9—H9C	0.9800
N4—C8	1.292 (3)		
			
N3—Fe1—N1	179.44 (10)	C1—C2—H2A	109.5
N3—Fe1—N2	98.66 (9)	C1—C2—H2B	109.5
N1—Fe1—N2	80.78 (9)	H2A—C2—H2B	109.5
N3—Fe1—N4	81.55 (9)	C1—C2—H2C	109.5
N1—Fe1—N4	99.01 (9)	H2A—C2—H2C	109.5
N2—Fe1—N4	179.79 (9)	H2B—C2—H2C	109.5
N3—Fe1—N5	92.39 (4)	O2—C3—N2	127.8 (3)
N1—Fe1—N5	87.63 (5)	O2—C3—C1	120.1 (2)
N2—Fe1—N5	91.64 (5)	N2—C3—C1	112.0 (2)
N4—Fe1—N5	88.35 (5)	N2—C4—C5	112.9 (2)
N3—Fe1—N5^i^	92.39 (5)	N2—C4—H4A	109.0
N1—Fe1—N5^i^	87.63 (4)	C5—C4—H4A	109.0
N2—Fe1—N5^i^	91.64 (5)	N2—C4—H4B	109.0
N4—Fe1—N5^i^	88.35 (5)	C5—C4—H4B	109.0
N5—Fe1—N5^i^	173.75 (9)	H4A—C4—H4B	107.8
N1—O1—H1O	104.8	C4—C5—C6	114.8 (3)
Fe1—N5—H5D	109.5	C4—C5—H5A	108.6
Fe1—N5—H5E	109.5	C6—C5—H5A	108.6
H5D—N5—H5E	109.5	C4—C5—H5B	108.6
Fe1—N5—H5F	109.5	C6—C5—H5B	108.6
H5D—N5—H5F	109.5	H5A—C5—H5B	107.5
H5E—N5—H5F	109.5	N3—C6—C5	111.8 (2)
C1—N1—O1	118.8 (2)	N3—C6—H6A	109.3
C1—N1—Fe1	118.60 (18)	C5—C6—H6A	109.3
O1—N1—Fe1	122.60 (16)	N3—C6—H6B	109.3
C3—N2—C4	119.4 (2)	C5—C6—H6B	109.3
C3—N2—Fe1	116.91 (18)	H6A—C6—H6B	107.9
C4—N2—Fe1	123.68 (17)	O3—C7—N3	127.0 (2)
C7—N3—C6	119.2 (2)	O3—C7—C8	120.8 (2)
C7—N3—Fe1	116.37 (17)	N3—C7—C8	112.2 (2)
C6—N3—Fe1	124.43 (17)	N4—C8—C9	124.4 (2)
C8—N4—O4^i^	121.3 (2)	N4—C8—C7	112.4 (2)
C8—N4—O4	121.3 (2)	C9—C8—C7	123.2 (2)
C8—N4—Fe1	117.49 (18)	C8—C9—H9A	109.5
O4^i^—N4—Fe1	121.24 (16)	C8—C9—H9B	109.5
O4—N4—Fe1	121.24 (16)	H9A—C9—H9B	109.5
N1—C1—C2	124.4 (3)	C8—C9—H9C	109.5
N1—C1—C3	111.7 (2)	H9A—C9—H9C	109.5
C2—C1—C3	123.9 (2)	H9B—C9—H9C	109.5
			
N5—Fe1—N1—C1	92.04 (4)	N5—Fe1—N4—C8	−92.66 (4)
N5^i^—Fe1—N1—C1	−92.04 (4)	N5^i^—Fe1—N4—C8	92.66 (4)
N5—Fe1—N1—O1	−87.96 (4)	N5—Fe1—N4—O4^i^	87.34 (4)
N5^i^—Fe1—N1—O1	87.96 (4)	N5^i^—Fe1—N4—O4^i^	−87.34 (4)
N5—Fe1—N2—C3	−87.34 (4)	N5—Fe1—N4—O4	87.34 (4)
N5^i^—Fe1—N2—C3	87.34 (4)	N5^i^—Fe1—N4—O4	−87.34 (4)
N5—Fe1—N2—C4	92.66 (4)	C3—N2—C4—C5	149.83 (18)
N5^i^—Fe1—N2—C4	−92.66 (4)	Fe1—N2—C4—C5	−30.17 (18)
N5—Fe1—N3—C7	87.98 (5)	N2—C4—C5—C6	66.1 (3)
N5^i^—Fe1—N3—C7	−87.98 (5)	C7—N3—C6—C5	−150.96 (17)
N5—Fe1—N3—C6	−92.02 (5)	Fe1—N3—C6—C5	29.04 (17)
N5^i^—Fe1—N3—C6	92.02 (5)	C4—C5—C6—N3	−65.3 (3)

Symmetry code: (i) *x*, −*y*+3/2, *z*.

### Hydrogen-bond geometry (Å, º)

**Table:**

*D*—H···*A*	*D*—H	H···*A*	*D*···*A*	*D*—H···*A*
O1—H1*O*···O4	0.98	1.54	2.508 (3)	168
N5—H5*D*···O4^ii^	0.91	2.15	3.015 (2)	158
N5—H5*E*···O2^iii^	0.91	2.11	2.979 (2)	160
N5—H5*F*···O3^iv^	0.91	2.10	2.969 (2)	160

Symmetry codes: (ii) −*x*+2, *y*+1/2, −*z*+1; (iii) −*x*+2, *y*+1/2, −*z*+2; (iv) −*x*+1, *y*+1/2, −*z*+1.
